# Dispositional Employability and Online Training Purchase. Evidence from Employees' Behavior in Spain

**DOI:** 10.3389/fpsyg.2016.00831

**Published:** 2016-06-01

**Authors:** Joan Torrent-Sellens, Pilar Ficapal-Cusí, Joan Boada-Grau

**Affiliations:** ^1^Faculty of Economics and Business, Open University of CataloniaBarcelona, Spain; ^2^Department of Psychology, Rovira i Virgili UniversityTarragona, Spain

**Keywords:** dispositional employability, online training, purchase, skills, career motivation, work resilience, work identity, Spain

## Abstract

This article explores the relationship between dispositional employability and online training purchase. Through a sample of 883 employees working for enterprises in Spain, and a using principal component analysis and binomial logit probabilistic models, the research revealed two main results. First, it was found that dispositional employability is characterized by five factors: “openness to changes at work,” “career motivation and work resilience,” “work and career proactivity,” “optimism and engagement at work,” and “work identity.” Second, the research also found a double causality in the relationship analysis between dispositional employability and online training purchase. However, this causality is not direct. In explaining dispositional employability, certain motivations and types of behavior of employees participating in online training are significant. In particular, greater sensitivity toward career-related personal empowerment, a greater predisposition toward developing new experiences at work, and a greater awareness of the fact that positive job outcomes are related to preparation conscientiousness. In explaining online training purchase, employees who are more motivated and who better identify with their jobs are more likely to pay. Moreover, employees who spend more time on training and have less contact with new trends in their jobs, find it hard to keep calm in difficult situations, and have a greater predisposition toward effort, and preference for novelty, variety and challenges at work are more likely to purchase online training.

## Introduction

In recent years, employability has become a growing field of economic, psychological, and social research (Thijssen et al., 2008; Smith, 2010; Hogan et al., 2013). It is acknowledged that employability is the ability to get and keep a job in a formal organization (Baruch, 2001; Harvey, 2001). The literature on employability emphasizes the important role of individual characteristics in adaptation at work. Ashford and Taylor (1990), and Ashford and Black (1996) claim that individual and psychological factors are essential components of effective adaptation at work. The premise is that employability is a synergistic collection of individual characteristics driven and directed by an individual's adaptability, career identity, and human and social capital (McArdle et al., 2007). Employability can be considered a psychosocial construct that enables career success (Fugate et al., 2004).

Within the psychological framework, empirical research on individual differences in career success represents the main contribution to the study of employability (Hogan et al., 2013). The literature on career success is typically organized into three combined models: views of human capital, of structure, and of social capital. According to the human capital view (Becker, 1975), organizations distribute rewards to their members according to their contributions, and employees' ability to contribute depends on having relevant skills and competing capabilities (Brown and Hesketh, 2004).

Although prior education plays a decisive role, the evidence suggests that the relationship between educational attainment and career success is only modest and acts as a “first-pass filter” (Pfeffer and Fong, 2002; Ng et al., 2005; Chamorro-Premuzic and Furnham, 2010). In this sense, and beyond the traditional relationship between cognitive ability and educational attainment, new research highlights the importance of personality characteristics and individuals' behavior (especially “effort,” “ability,” “curiosity,” and “conscientiousness”) as predictors of not only academic performance (Ng and Feldman, 2010; von Stumm et al., 2011) but also job outcomes (Roberts et al., 2007). Economic research has also obtained similar results (Lindqvist and Westman, 2011).

In contrast with the large body of research on the psychological determinants of career success, there has been little research on the determinants of employability (Baruch and Bozionelos, 2011). According to the literature, employability depends on identifiable personal characteristics that can be assessed and possibly trained (Fugate et al., 2004; Smith, 2010). Van der Heijde and Van der Heijden (2006) propose a five-scale employability model: “occupational expertise” (job competences), “anticipation” (job ambition), “personal flexibility” (high degree of openness, conscientiousness, and adjustment), “corporate sense” (disposition to behave in a social manner), and “balance” (work-life proportionality). Their best employability predictor was “corporate sense.” Thijssen et al. (2008) propose an employability model that includes three dimensions: an individual's “ability to perform a job,” “personal skills and learning capabilities,” and “contextual factors” (organizational and social), which may influence future employment status. Finally, Wittekind et al. (2009) consider three employability dimensions: “job-related skills,” “willingness to learn and develop new skills,” and “knowledge of the labor market.” Like career success, employability seems to be more a function of personal characteristics and behavior than of educational level.

Based on this evidence, a self-perceived employability approach (Ghoshal et al., 1999; Forstenlechner et al., 2014) has been developed in the literature. Perceived employability refers to an individual's perception of his or her possibilities of getting and keeping a job (Vanhercke et al., 2014). It is an individual's subjective evaluation of such possibilities and involves the integration of personal and structural (job, organizational, and social) characteristics (Berntson and Marklund, 2007). Consequently, perceived employability is relevant to different groups in the labor market throughout their careers. Evidence on perceived employability has been obtained from graduate students (Rothwell et al., 2009), employees (Rothwell and Arnold, 2007), and the unemployed (Wanberg et al., 2010).

Perceived employability can be applied to either current employment (internal labor markets) or future jobs (external labor markets) (Eby et al., 2003). Finally, it also concerns a focus upon both the quantity and quality of jobs (De Cuyper and De Witte, 2010). The literature shows that self-perceived employability is a robust measure across cultures (Rothwell et al., 2009) and is positively related to self-determination. Self-determination refers to a combination of skills, knowledge, and beliefs that enable employees to make decisions and re-evaluate past solutions, to generate new solutions if needed, and choose the best option to engage in goal-directed self-regulated behavior (Parker et al., 2010).

In the literature, two additional approaches have been taken to the explanation of perceived employability: the competence-based approach and the dispositional approach (De Cuyper et al., 2012). The competence-based approach focuses on an individual's perception of his/her abilities, capacities, and skills (specific or generic competences) that promote employment opportunities (Van der Heijde and Van der Heijden, 2006). The dispositional approach focuses on the perception of an individual's proactive attitudes toward his/her career and work in general (Fugate and Kinicki, 2008).

When comparing the three approaches, several similarities can be found. Firstly, all three approaches focus on an individual's perceptions: the labor market position in the perceived employability approach, employability abilities in the competence-based approach, and motivational attitudes in the dispositional employability approach. Also, all three approaches account for personal and structural factors, as well as their interaction, in explaining internal or external employability. Regarding the differences between the three approaches, it is important to note that while the perceived employability approach has been applied to different groups in the labor market and across career stages, the competence-based and dispositional approaches have been applied mainly to employees. Also, the perceived employability approach endorses both the quantity and quality of jobs, while this distinction is less relevant for the competence-based and dispositional approaches.

The flexibilization, segmentation, and individualization of labor markets, the advent of new work organization and human resources practices, and the global knowledge-based economy have fostered new approaches to the labor market in general (Torrent-Sellens and Ficapal-Cusí, 2009) and to employability research in particular (Brown et al., 2003). Some studies have noted the influence of the labor market's growing flexibility and the materialization of new contracts and labor relations frameworks (Esser and Olsen, 2012). Also noted in the literature is the emergence of new problems associated with structural change in employment (Green and Mostafa, 2012). From the point of view of new employability trends, recent research has also made significant progress. Specifically, it has highlighted the importance of employees' future job expectations, which are clearly linked to educational level (Gallie et al., 2012). Workers with a highly positive perception of the future of their jobs tend to have better employability and better quality jobs (Graso and Probst, 2012).

In the global knowledge economy, where technology and innovation are key to developing enterprise competitiveness, new value generation processes and sources of productivity inevitably call for trending changes in employability (Díaz-Chao et al., 2015, 2016a). In such an economy, creating and maintaining employment depends largely on employability factors, of enterprises' ability to generate jobs with trained, autonomous, committed and satisfied employees, who are able to innovate and create more added value (Díaz-Chao et al., 2016b).

Thus, within the context of changes in employability resulting from the advent of the knowledge-based economy, this article analyzes the determinants thereof. In this regard, it is important to note the following considerations. First, according to the literature on employability, our perspective goes beyond the traditional relationship between educational attainment and employability to take into account employees' motivations and behavior. Second, in accordance with the literature on knowledge-based employment, our perspective bears in mind the relevance of motivational attitudes toward new job-related trends. Third, as discussed below, we consider a specific form of training that has proved to be very important in the knowledge economy: online training. And fourth, we work with employees' motivational perceptions and with personal and structural factors. We therefore take a dispositional employability approach, in which we understand employability as “a constellation of individual differences that predispose employees to (pro)actively adapt to their work and career environments” (Fugate and Kinicki, 2008, p. 504). This perceived employability approach is reflected in five dimensions: “openness to changes at work,” “work and career resilience,” “work and career proactivity,” “career motivation,” and “work identity.” Our first research hypothesis is:

H1. Employees who are more motivated to anticipate and optimize job changes, who identify with and are involved in corporate sense, who have less stress, who have self-efficacy or the ability to manage obstacles, overcome difficulties and achieve their goals, and who have hardy personality traits to commit, challenge, and control job situations are more likely to have greater dispositional employability.

As a result of the advent of the knowledge-based economy, the restructuring of business activity has also transformed the foundations of labor. The impact of knowledge-based innovation on the organization, conditions, and results of labor remains an open debate in the literature (Osterman, 2000; Neumark and Reed, 2004). On the one hand, the introduction of information and communication technologies (ICT) and massive flows of knowledge have gone hand in hand with an increase in cognitive demands, enhanced autonomy, a decline in hierarchical control, a rise in job creation and maintenance, and improvements in wages. But, on the other hand, it has led to a decline in labor based on routine manual and routine cognitive tasks, and the downskilling and destruction of employment in some segments of the population or industries (Autor et al., 2003).

In the context of labor market transformation, the debate on continuing training -particularly in online environments- acquires special importance in the sense that continued learning processes are placed at the heart of career development and the improvement of employees' employability (De Vos et al., 2011). Although the positive relationship between educational attainment and employability in the knowledge-based economy has already been amply contrasted in the empirical literature on human capital (Heckman, 2004), in recent years an employability training divide has also been confirmed. In this regard, vocational education and training, understood as on-the-job learning activities, are mostly done by people who are already trained (Kyndt and Baert, 2013). One of the reasons that might explain this phenomenon is the lack of suitability between educational and training programs for employability and the socio-cultural complexity of jobs that are constantly changing (Billet and Choy, 2013). Also noted in the literature is the importance of certain student characteristics and types of behavior, such as their attitudes toward their careers and their self-determination, which would be clearly linked to their prior educational level (Kyndt and Baert, 2013).

Indeed, and despite their significant potential, the emergence, and consolidation of online training programs for employability has not managed to break down this educational barrier. The empirical evidence shows us, again, that online training for employability encounters problems when it comes to training those who are not trained. As is the case with non-online training, the use of learning methodologies that are not very collaborative, are undertaken outside the workplace, and are poorly adapted to the socio-cultural reality and specific characteristics of the students seems to explain the weak results of online training for employability (Inayat et al., 2013; Silva et al., 2013). In this sense, our second research hypothesis relates to the motivational and behavioral issues of employees performing online training activities:

H2. The effect of online training on dispositional employability is indirect. Employees participating in online training programs who are more sensitive to career-related personal empowerment, more predisposed to developing new experiences at work, and more aware of the fact that positive job outcomes are related to preparation conscientiousness are more likely to have greater dispositional employability.

Finally, and beyond their technological and pedagogical dimensions (Selim, 2007; Sun et al., 2008; Lee et al., 2009), employees' personal, and motivational determinants of online training enrolment and satisfaction (Rosenberg, 2001) have also been addressed in the literature. Regarding socio-demographic determinants, gender, and age differences in the effects of e-learning, including students' satisfaction and Internet self-efficacy, have been supported in prior research (Chen and Tsai, 2007). In this sense, new research has confirmed that emotional family support has both direct and indirect influences on students' perceived effects of e-learning (Chu, 2010). Tangible support significantly predicts adults' perceived effects of e-learning, mediated by Internet self-efficacy. Compared to male adult learners, female adult learners rely more on tangible family support for increasing their Internet self-efficacy. The similarities between women and older adults imply that the issue is not specifically related to gender, but instead relates to the complexity of the social context of these disadvantaged learners.

Regarding the motivational and behavioral dimension, a set of determinants related to job features has also been identified in the literature. Brown (2005) shows that employees' perceptions of peer and supervisor support, job characteristics (especially workload and autonomy), and motivation to learn predict time spent on online training courses. In a comparative study, Rovai et al. (2007) provide evidence that e-learning students possess stronger intrinsic motivation than on-campus students who attend face-to-face classes on three intrinsic motivation measures: to know, to accomplish things, and to experience stimulation. In a learner loyalty approach, Chiu and Wang (2008) indicated that performance expectancy, effort expectancy, computer self-efficacy, attainment value (self-image and core personal values), utility value (current and future career goals), and intrinsic value (personally enjoyable activity) were significant predictors of students' intentions to continue using Web-based learning, while anxiety had a significant negative effect. In a perceived learning achievements approach, Paechter et al. (2010) show that students who attach a high value to specific achievements are also likely to invest more effort in learning, to apply more elaborate information processing strategies, or to dedicate more time to learning. In addition, flexibility in the choice of learning strategies and the exchange of innovative knowledge with peer students are positively related to learning achievements. Compiling this evidence, our third research hypothesis is:

H3. Employees are more likely to purchase online training if they are younger, women, more motivated, better identify with their jobs, have more time to spend on training, do not have recent contact with new trends in their jobs, find it hard to keep calm in difficult skill situations, have a greater predisposition toward effort, and preference for novelty, variety, and challenges at work.

## Materials and methods

### Sample

The sample was selected by random accidental sampling (Kerlinger, 2001). The response rate was 83.7%. Of the original sample, 12.2% of the employees abstained from participating, and a further 4.1% of the questionnaires returned were rejected due to completion mistakes or omissions. After contacting the employees selected to take part in the study, the Web-based interviews were administered individually in work time with the prior consent of the enterprises' managers. The participants were given instructions to enable them to answer the questionnaires. They were also given an assurance about the confidentiality and anonymity of the data obtained. The fieldwork period ran from 15 May to 4 June 2013.

This research was mainly designed and performed as a research work in the Open University of Catalonia (UOC)—the institution where the first and second author works. In the following link you will be able to find the function and aims of the UOC ethics committee: www.uoc.edu/portal/en/recerca-innovacio/activitat-rdi/comite-etica/funcions/index.html. No permission from a board or committee is necessary in the case of own lecturers social sciences research. In the context of social sciences (and not only in the case of this university) voluntary completion of questionnaires after the goals of the research are explained, is considered as a guarantee that individuals want to participate in the study.

The sample comprised 833 employees working for enterprises operating in Spain. Of the total, 49.8% was male and 50.2% female. The employees' mean age was 34.3 years (*SD* = 8.83), distributed in the following range: 18–25 years (15.5%), 26–34 years (41.1%), 35–45 years (30.1%), and 46–65 years (13.3%). The participants were highly educated. Most of them had completed at least a bachelor's degree (72.5%). The mean length of work experience was 10.4 years (*SD* = 8.14) in their workplace and 7.4 years (*SD* = 7.37) in their enterprises. The employees belonged to enterprises whose activities covered a wide sectoral range, such as financial intermediation, education and social services, health and hospitals, commerce, telecommunications, metallurgy and other similar activities, pharmaceuticals, chemicals, security, sales-oriented services, information, and communication technologies (ICT), general consultancy, hotel industry, distribution, tourism, and food.

### Measures

We measured dispositional employability by adapting the five-factor scale created by Fugate and Kinicki (2008). In their study, the first factor “openness to changes at work” (α = 0.76) comprises five items; the second factor “work and career resilience” (α = 0.75) comprises eight items; the third factor “work and career proactivity” (α = 0.55) comprises three items; the fourth factor “career motivation” (α = 0.68) comprises three items, and the fifth factor “work identity” (α = 0.66) comprises six items. The employees answered the questions on a five-point Likert scale, where: 1 = never/none; 2 = sometimes/little; 3 = usually/somewhat; 4 = almost always/quite; and 5 = always/a lot.

In order to obtain the predictors of dispositional employability and online training purchase, we used items for three additional and adapted scales. The first one uses a competence-based approach to employability (Van der Heijde and Van der Heijden, 2006). Five factors of employability are considered: “occupational expertise” (α = 0.90) comprises 15 items; “anticipation and optimization” (α = 0.81) comprises eight items; “personal flexibility” (α = 0.79) comprises eight items, “corporate sense” (α = 0.83) comprises seven items and “balance” (α = 0.78) comprises nine items. The employees answered the questions on a five-point Likert scale, where: 1 = never/none; 2 = sometimes/little; 3 = usually/somewhat; 4 = almost always/quite; and 5 = always/a lot.

The second one is the General Self-Efficacy Scale (Baessler and Schwarcer, 1996). Self-efficacy refers to people's firm belief in their ability to appropriately manage a wide range of obstacles and adverse experiences (Bandura, 1997, 1986). This is a one-dimensional construct explained through 10 items (α = 0.81), and measures employees' opinions on a Likert-scale from 1 to 10: 1 = Totally disagree and 10 = Totally agree. The third one is the Hardy Personality Scale (Moreno-Jiménez et al., 2001). The Hardy personality construct (Kobasa, 1979) refers to the personality model of an individual who, when active and committed, perceives stress stimuli as less threatening. The scale is formed by three factors: “commitment” (α = 0.81) comprises eight items; “control” (α = 0.75) comprises six items; and “challenge” (α = 0.81) comprises seven items. The employees answered the questions on a Likert-scale from one to four: 1 = Totally disagree; 2 = Somewhat disagree; 3 = Somewhat agree; 4 = Totally agree.

### Procedure

The dispositional employability indicator was constructed by following the steps shown in the literature (Muñiz and Bartram, 2007; Muñiz et al., 2013). First, the initial items were translated from English into Spanish by research experts (university lecturers) and language experts belonging to the Language Service at the UOC in Spain. Second, a focus group was held to discuss the translated items (equivalence of meaning, for example). Third, the language experts back-translated the items into English. Fourth and lastly, the equivalence of meaning of the original and adapted versions was checked.

In order to build the exploratory factor analysis structure and the reliability coefficients of the dispositional employability measure, the FACTOR 7.2 program was used (Lorenzo and Ferrando, 2006). This program allows exploratory analysis to be performed using polychoric correlation matrices. It also allows additional analyses—unavailable in SPSS 21.0—to be performed, such as parallel analysis. SPSS 21.0 was used to evaluate the internal consistency of the dispositional employability scale (alpha coefficients). An exploratory factor analysis was therefore performed to analyze the dimensionality of the dispositional employability measure by applying the principal axis extraction and oblimin rotation methods. In addition, polychoric correlation matrices were used; these are particularly suitable for items with a Likert-type response format (Lorenzo and Ferrando, 2009). Table 1 presents the descriptive statistics of the items obtained from the exploratory factor analysis.

**Table 1:** **Dispositional employability items: mean, standard deviation, Skewness, and Kurtosis**.

	**Mean**	***SD***	**Min**.	**Max**.	**Sk**.	**Kur**.
**OPENNESS TO CHANGES AT WORK**						
- I feel changes at work generally have positive implications	3.36	0.98	1	5	−0.07	−0.83
- I feel that I am generally accepting of changes at work	3.81	0.89	1	5	−0.51	−0.15
- I would consider myself open to changes at work	3.85	0.89	1	5	−0.56	0.00
- I can handle job and organizational changes effectively	3.73	0.84	1	5	−0.45	0.17
- I am able to adapt to changing circumstances at work	3.88	0.86	1	5	−0.63	0.31
**CAREER MOTIVATION AND WORK RESILIENCE**						
- I am optimistic about my future career opportunities	3.55	1.11	1	5	−0.39	−0.63
- In uncertain times at work, I usually expect the best	3.18	1.04	1	5	−0.03	−0.61
- I have a specific plan for achieving my career goals	3.44	1.12	1	5	−0.32	−0.72
- I have sought job assignments that will help me obtain my career goals	3.52	1.09	1	5	−0.39	−0.64
- I have control over my career opportunities	3.35	1.04	1	5	−0.25	−0.60
- I am a believer that “every cloud has a silver lining” at work	3.20	1.08	1	5	−0.04	−0.60
**WORK AND CAREER PROACTIVITY**						
- I stay abreast of developments in my enterprise	3.95	0.94	1	5	−0.73	0.16
- I have participated in training or schooling that will help me reach my career goals	3.69	1.20	1	5	−0.67	−0.46
- I stay abreast of developments in my industry	3.74	0.96	1	5	−0.61	0.03
- I stay abreast of developments relating to my type of job	3.73	0.93	1	5	−0.54	0.06
**OPTIMISM AND ENGAGEMENT AT WORK**						
- I always look on the bright side of things at work	3.62	0.92	1	5	−0.31	−0.23
- I am involved in my work	4.43	0.74	1	5	−1.31	1.68
- My past career experiences have been generally positive	3.88	0.92	1	5	−0.73	0.32
- I take a positive attitude toward my work	4.01	0.87	1	5	−0.68	0.14
- The type of work I do is important to me	3.97	0.99	1	5	−0.82	0.11
**WORK IDENTITY**						
- It is important to me that others think highly of my job	4.25	0.84	1	5	−1.22	1.67
- It is important to me that I am successful in my job	4.27	0.83	1	5	−1.07	0.88
- It is important to me that I'm acknowledged for my jobs' successes	4.04	0.96	1	5	−0.99	0.75

*N = 883*.

After obtaining the factors of dispositional employability, we performed a probabilistic analysis of discrete choice (binomial logit). We dichotomized the factors and the employability composite indicator by their mean values. We then identified the factors explaining greater propensity toward dispositional employability. In the same way, we explored the factors explaining online training purchase. The interpretation of standardized coefficients determines the probability of independent variables explaining Spanish employees' greater dispositional employability and online training purchase.

In order to estimate the overall effect of individual variables on the explanation of dispositional employability, we applied a binomial logit model. The parameters for the binary logistic regression model are described below. Dispositional employability is the dichotomous dependent variable (DDISEMP). It takes value 1 when the composite indicator of dispositional employability (an arithmetic mean of the factors obtained in the exploratory factor analysis) are equal to or greater than the mean, and value 0 otherwise. This dichotomous indicator has a mean value of 0.5 points and a standard deviation of 0.4 points. Some 52.1% of Spanish employees' dispositional employability is higher (greater than the mean).

Regarding the independent variables, we contemplated a first group of four discrete explanatory variables related to job ambition, in the sense of anticipating and optimizing job changes. Self-initiating proactive job ambition entails employees preparing for future work changes in order to strive for the best possible job and career outcomes. The TRAINING variable relates to vocational education and training and responds to the item: “Time dedicated to improving the knowledge and skills that will be useful at work.” The CORRECTING variable relates to the systematic correction of job weaknesses and responds to the item: “I try to correct my weaknesses in a systematic way.” The NTRENDING variable relates to interacting with new job-related trends and responds to the item: “Last year, I interacted with the latest developments and trends in my job.” The PERSEMPOWR (Personal Empowerment) variable relates to employee responsibility for assessing their job value and responds to the item: “I take responsibility for keeping my value in the job market.” These four variables are taken from the employability competence-based approach from Van der Heijde and Van der Heijden (2006).

The analysis also incorporates two additional variables relating to job competences. The first relates to organizational identification, in terms of corporate sense. In new working environments, employees have to participate more as members of an integrated team, identify with corporate goals and accept collective responsibility for the decision-making process. The variable PARTICIPATION relates to employees' participation in corporate sense, and responds to the item: “In my organization, I participated in the formation of a vision with common values and objectives.” The second relates to work-life balance. The STRESS variable relates to employees' stress at work and responds to the item: “I suffer job-related stress.”

A second group of three discrete explanatory variables is associated with self-efficacy and work-related behavior. Self-efficacy refers to employees' ability to appropriately manage a wide range of obstacles, overcome difficulties and achieve their goals. The PERSISTENCE variable relates to the capacity for persistence and responds to the item: “I find it easy to persist in what I have set up to achieve my goals.” The OVERCOME variable relates to the ability to overcome unexpected situations and responds to the item: “Thanks to my skills and resources, I can overcome unexpected situations.” And, the CALM variable relates to the ability to keep calm in difficult work situations and responds to the item: “When I'm in trouble, I can stay calm because I have the necessary skills to handle difficult situations.” These three variables are taken from the General Self-Efficacy Scale (Baessler and Schwarcer, 1996).

And finally, a third group of four discrete explanatory variables is associated with a hardy personality and work-related behavior. Employees with hardy personalities are characterized by a high degree of commitment, experiencing situations as challenges rather than as threats, and tend to perceive a certain degree of internal control in various situations (Jiménez et al., 2006). The CONSCIENTIOUSNESS variable relates to preparation for positive job outcomes and responds to the item: “Things go well when you prepare thoroughly.” The NEXPERIENCE variable relates to a job choice involving new experiences and responds to the item: “Even if that entails more effort, I choose the work involving a new experience for me.” The INNOVATIVENESS variable relates to the preferences for innovative jobs and procedures, and responds to the item: “In my job, I preferably attract innovations and new developments in the proceedings.” And, the COMMITMENT variable relates to employees' commitment and responds to the item: “My daily work satisfies me and makes me totally dedicated to it.” These four variables are taken from the Hardy Personality Scale (Moreno-Jiménez et al., 2001).

Table 2 presents the descriptive statistics of the dichotomous dispositional employability explanatory and discrete Likert-point variables. The analysis of the correlation matrix between explanatory variables suggests the absence of multicollinearity (correlations < 0.4 points).

**Table 2:** **Dichotomous dispositional employability explanatory variables: mean, standard deviation, Skewness, and Kurtosis**.

	**Mean**	***SD***	**Min**.	**Max**.	**Sk**.	**Kur**.
**JOB AMBITION**						
- Time dedicated to improving the knowledge and skills that will be useful at work (TRAINING)	3.45	0.99	1	5	−0.32	−0.49
- I try to correct my weaknesses in a systematic way (CORRECTING)	3.69	0.89	1	5	−0.41	−0.08
- Last year, I interacted with the latest developments and trends in my job (NTRENDING)	3.46	1.00	1	5	−0.38	−0.32
- I take responsibility for keeping my value in the job market (PERSEMPOWR)	4.02	0.92	1	5	−0.86	−0.47
**ORGANIZATIONAL IDENTIFICATION**						
- In my organization, I participated in the formation of a vision with common values and objectives (PARTICIPATION)	3.49	1.03	1	5	−0.35	−0.42
**WORK BALANCE**						
- I suffer job-related stress (STRESS)	3.04	1.13	1	5	0.16	−0.94
**SELF-EFFICACY AND WORK-RELATED BEHAVIOR**						
- I find it easy to persist in what I have set up to achieve my goals (PERSISTENCE)	7.60	1.83	1	10	−0.96	1.16
- Thanks to my skills and resources, I can overcome unexpected situations (OVERCOME)	7.58	1.51	1	10	−0.65	0.78
- When I'm in trouble, I can stay calm because I have the necessary skills to handle difficult situations (CALM)	6.86	1.72	1	10	−0.54	0.18
**HARDY PERSONALITY AND WORK-RELATED BEHAVIOR**						
- Things go well when you prepare thoroughly (CONSCIENTIOUSNESS)	3.40	0.68	1	4	−0.84	0.14
- Even if that entails more effort, I choose the work involving a new experience for me (NEXPERIENCE)	3.24	0.70	1	4	−0.49	−0.43
- In my job, I preferably attract innovations and new developments in the proceedings (INNOVATIVENESS)	3.11	0.78	1	4	−0.47	−0.50
- My daily work satisfies me and makes me totally dedicated to it (COMMITMENT)	2.90	0.81	1	4	−0.35	−0.41

*N = 883*.

Completing the previous analysis, and in order to estimate the overall effect of individual variables on the explanation of paid online training, we re-applied a binomial logit model. The parameters for the binary logistic regression model are described below. Paid online training is the dichotomous dependent variable (PONLINET). It takes value 1 when employees participate in paid online training activities and value 0 otherwise. This dichotomous indicator has a mean value of 0.52 points and a standard deviation of 0.5 points. Some 52.2% of Spanish employees are in paid online training.

Regarding the independent variables, we contemplated a first group of two explanatory variables related to the socio-demographic conditions. The AGE variable relates to the age of employees. The variable is discrete and takes four values: 18–25 years (value 1), 26–34 years (value 2), 35–45 years (value 3), and 46–65 years (value 4). The GENDER variable relates to the gender of employees. The variable is dichotomous and takes the value 1 for men and value 0 for women.

A second group of two explanatory variables related to dispositional employability factors. The MOTIVATION variable refers to “career motivation and work resilience” factor obtained from the exploratory factor analysis (F2). This factor specifically relates to optimism and control over career opportunities, and assigning and planning career goals. The IDENTITY variable refers to the work identity factor also obtained from the exploratory factor analysis (F5). This factor specifically relates to the importance of success and external acknowledgment of work. Both factors have been dichotomized according to their mean values: value 1 when the factors are equal to or greater than the mean; and value 0 when they are less than the mean.

A third group of two discrete explanatory variables related to job ambition (i.e., anticipation and optimization of job changes) from the competence-based approach to employability (Van der Heijde and Van der Heijden, 2006). The TRAINING variable relates to vocational education and training and responds to the item: “Time dedicated to improving the knowledge and skills that will be useful at work.” The NTRENDING variable relates to employees interacting with new job-related trends and responds to the item: “Last year, I interacted with the latest developments and trends in my job.”

The fourth group of one discrete explanatory variable related to employees' self-efficacy or the ability to achieve their goals and overcome difficulties (Baessler and Schwarcer, 1996). The CALM variable relates to the ability to keep calm in difficult work situations and responds to the item: “When I'm in trouble, I can stay calm because I have the necessary skills to handle difficult situations.”

The fifth and final group of four discrete explanatory variables is associated with the hardy personality traits of employees who, when active and committed, react better to stressful stimuli at work (Moreno-Jiménez et al., 2001). The EFFORT variable relates to job effort and responds to the item: “Things are only based on personal effort.” The CHALLENGE variable relates to preferences for job challenges and responds to the item: “In my work I attract those tasks and situations involving a personal challenge.” The VARIETY variable relates to employees' preferences for job variety and responds to the item: “I like the fact that there is great variety in my work.” The NOVELTY variable relates to job choice involving new and different job situations, and responds to the item: “As far as possible, I seek new and different situations in my working environment.”

Table 3 presents the descriptive statistics of the dichotomous online training purchase explanatory and discrete variables. The analysis of the correlation matrix between the explanatory variables suggests the absence of multicollinearity (correlations < 0.4 points).

**Table 3:** **Online training purchase explanatory variables: mean, standard deviation, Skewness, and Kurtosis**.

	**Mean**	***SD***	**Min**.	**Max**.	**Sk**.	**Kur**.
**SOCIO-DEMOGRAPHICS CONDITIONS**						
- AGE: (1 = 18–25 years; 2 = 26–34 years; 3 = 35–45 years; 4 = 46–65 years)	2.41	0.91	1	4	0.17	0.16
- GENDER: (1 = man; 0 = woman)	0.50	0.50	0	1	0.00	−2.00
**DISPOSITIONAL EMPLOYABILITY FACTORS**						
- Career motivation and work resilience (MOTIVATION: 1 = equal or greater than the mean; 0 = less than the mean)	0.52	0.20	0	1	−0.10	−1.99
- Work identity (IDENTITY: 1 = equal or greater than the mean; 0 = less than the mean)	0.53	0.20	0	1	−0.12	−1.99
**JOB AMBITION**						
- Time dedicated to improving the knowledge and skills that will be useful at work (TRAINING)	3.45	0.99	1	5	−0.32	−0.49
- Last year, I interacted with the latest developments and trends in my job (NTRENDING)	3.46	1.00	1	5	−0.38	−0.32
**SELF-EFFICACY AND WORK-RELATED BEHAVIOR**						
- When I'm in trouble, I can stay calm because I have the necessary skills to handle difficult situations (CALM)	6.86	1.72	1	10	−0.54	0.18
**HARDY PERSONALITY AND WORK-RELATED BEHAVIOR**						
- Things are only based on personal effort (EFFORT)	3.40	0.79	1	4	−1.16	−0.57
- In my work I attract those tasks and situations involving a personal challenge (CHALLENGE)	3.31	0.70	1	4	−0.65	−0.23
- I like the fact that there is great variety in my work (VARIETY)	3.36	0.71	1	4	−0.82	0.03
- As far as possible, I seek new and different situations in my working environment (NOVELTY)	2.98	0.78	1	4	−0.27	−0.57

*N = 883*.

## Results

### Dispositional employability indicator

The results of Bartlett's sphericity test (Chi-square = 7110.9; *p* < 0.01) and the Kaiser-Meyer-Olkin index of sampling adequacy (KMO = 0.898) confirmed the adequacy of the data for factor analysis. The scree test, parallel analysis, and the minimum average partial test yielded a solution based on five factors (Timmerman and Lorenzo, 2011).

After establishing the most suitable factor solution, the oblimin rotation method was used to obtain a simple factor solution. This method of oblique rotation tends to yield the very simplest of solutions, even in cases where one of the items displays a complex structure. The scale was honed down from the 25 original items by removing any that had saturations lower than 0.4, or complex saturations higher than 0.4 in more than one factor. The resulting number of items with the highest saturations was 23, distributed as follows: five items for the “openness to changes at work” factor, six items for the “career motivation and work resilience” factor, four items for the “work and career proactivity” factor, five items for the “optimism and engagement at work” factor, and threeitems for the “work identity” factor.

The saturation matrix of the factor solution obtained enabled us to identify the content of the five factors, which together explained 56.4% of cumulative variance. The correlation between the five factors was rather high; it varied between 0.31 and 0.61. The α-reliability for the combined scale was 0.75.

The first factor obtained (F1) refers to “openness to changes at work” (Cronbach's alpha = 0.72). This factor specifically relates to employees' positive feelings, openness, adaptation, management, and acceptance of changes at work. It explains 30.3% of the variance, and has a mean of 0.1 points and a standard deviation of 0.9 points. The second factor (F2) refers to “career motivation and work resilience” (Cronbach's alpha = 0.75), which explains 8.1% of the variance, and has a mean of 0.6 points and a standard deviation of 1.1 points. This factor specifically relates to optimism and control over career opportunities, and assigning and planning career goals. The third factor (F3) refers to “work and career proactivity” (Cronbach's alpha = 0.80). This factor specifically relates to employees' ability to keep abreast (including vocational education and training) of new developments in their jobs, enterprises and industries. It explains 7.2% of the variance, and has a mean of 0.7 points, and a standard deviation of 1.1 points.

The fourth factor (F4) refers to “optimism and engagement at work” (Cronbach's alpha = 0.76), which explains 6% of the variance, and has a mean of 0.1 points and a standard deviation of 0.8 points. This factor specifically relates to employees' positive thinking, attitudes and involvement in work. Finally, the fifth factor (F5) refers to “work identity” (Cronbach's alpha = 0.70). This factor specifically relates to the importance of success and external acknowledgment of work. It explains 4.8% of the variance, and has a mean of 0.9 points and a standard deviation of 1.2 points. Table 4 shows the factor scores, reliability coefficients, variance explained, mean, standard deviation, and correlations between the five factors obtained from the exploratory factor analysis. Items in the Spanish language are also presented.

**Table 4:** **Dispositional employability indicator: Factor loadings, explained variance, and correlations**.

	α	1	2	3	4	5
Factor 1—Openness to changes at work	0.72					
- I feel changes at work generally have positive implications [Creo que, en general, los cambios en el trabajo tienen implicaciones positivas]		0.55				
- I feel that I am generally accepting of changes at work [Creo que, en general, acepto los cambios en el trabajo]		0.78				
- I would consider myself open to changes at work [Me considero una persona abierta a los cambios en el trabajo]		0.79				
- I can handle job and organizational changes effectively [Gestiono con eficacia los cambios en el trabajo]		0.67				
- I am able to adapt to changing circumstances at work [Soy capaz de adaptarme a unas circunstancias cambiantes en el trabajo]		0.79				
Factor 2—Career motivation and work resilience	0.75					
- I am optimistic about my future career opportunities [Soy optimista sobre mis oportunidades profesionales en el futuro]			0.62			
- In uncertain times at work, I usually expect the best [En tiempos de Incertidumbre en el trabajo, normalmente espero lo mejor]			0.66			
- I have a specific plan for achieving my career goals [Tengo un plan concreto para alcanzar mis objetivos profesionales]			0.65			
- I have sought job assignments that will help me obtain my career goals [He buscado que me asignen tareas que me ayuden a alcanzar mis objetivos profesionales]			0.55			
- I have control over my career opportunities [Controlo mis oportunidades profesionales]			0.65			
- I am a believer that “every cloud has a silver lining” at work [En el trabajo, creo que no hay mal que por bien no venga]			0.56			
Factor 3—Work and career proactivity	0.8					
- I stay abreast of developments in my enterprise [Me mantengo al día de los cambios en mi empresa]				0.59		
- I have participated in training or schooling that will help me reach my career goals [He participado en cursos o formación que me ayudarán a alcanzar mis objetivos profesionales]				0.50		
- I stay abreast of developments in my industry [Me mantengo al día de los cambios en mi sector]				0.79		
- I stay abreast of developments relating to my type of job [Me mantengo al día de los cambios relacionados con mi tipo de empleo]				0.74		
Factor 4—Optimism and engagement at work	0.76					
- I always look on the bright side of things at work [En el trabajo, siempre miro el lado bueno de las cosas]					0.54	
- I am involved in my work [Me implico en mi trabajo]					0.54	
- My past career experiences have been generally positive [En general, mis experiencias profesionales pasadas han sido positivas]					0.49	
- I take a positive attitude toward my work [Mantengo una actitud positive hacia mi trabajo]					0.72	
- The type of work I do is important to me [El tipo de trabajo que hago es importante para mí]					0.63	
Factor 5—Work identity	0.7					
- It is important to me that others think highly of my job [Para mí es importante que otras personas tengan una buena opinión de mi trabajo]						0.71
- It is important to me that I am successful in my job [Para mí es importante tener éxito en mi trabajo]						0.79
- It is important to me that I am acknowledged for my successes in the job [Para mi es importante ser reconocido/a por mis éxitos en el trabajo]						0.84
% explained variance	–	30.29	8.12	7.24	6.02	4.77
Mean	–	0.11	0.61	0.67	0.12	0.86
Standard deviation	–	0.85	1.14	1.17	0.79	1.22
F2	–	0.61^*^	–	–	–	–
F3	–	0.46^*^	0.43^*^	–	–	–
F4	–	0.31^*^	0.32^*^	0.59^*^	–	–
F5	–	0.42^*^	0.46^*^	0.55^*^	0.51^*^	–

* *p < 0.01. N = 883*.

### Dispositional employability explanatory factors

The probabilistic model correctly classified 81.3% of employees (79.0% of those who do not have a greater disposition toward employability and 83.5% of those who have a greater disposition toward employability). The Cox-Snell and Nagelkerke *R*^2^-values were 0.43 and 0.58, respectively. The improvement (from 500.5 to 722) in the likelihood function is significant. It confirms the goodness-of-fit of the predictive capacity, and the variables as a whole have an outstanding explanatory power (Chi-square Hosmer-Lemeshow test = 15.1; *p* = 0.047).

From the model's estimation (Table 5), it was found that all the included variables have significant explanatory power for Spanish employees' greater disposition toward employability (*p* < 0.1 in the worst case). Regarding standardized coefficients and odds ratios [Exp (β)], the variables with greater explanatory power are employees' career-related personal empowerment, participation in corporate sense, correcting job weaknesses, and commitment to employment. In this sense, it is confirmed that the predisposition toward more employability would be explained differentially by three dimensions linked to employees' behavior to anticipate and optimize their job changes or job ambition, to identify and participate in corporate sense, and by the hardy personality traits linked with the commitment to employment.

**Table 5:** **Determinants of the dispositional employability**.

	**Standardized coefficient**	**Standard error**	**Wald**	**Exp (β)**	**95% CI**	
					**Lower**	**Higher**
**ALL EMPLOYEES (*****N*** = **883)**						
Constant	−16.595^***^	1.257	174.179	–	–	–
**Job Ambition**						
TRAINING	0.301^**^	0.109	7.560	1.351	1.090	1.673
CORRECTING	0.679^***^	0.123	30.275	1.972	1.548	2.512
NTRENDING	0.291^**^	0.109	7.115	1.338	1.080	1.656
PERSEMPOWR	0.779^***^	0.120	42.319	2.179	1.723	2.755
**Organizational Identification**						
PARTICIPATION	0.695^***^	0.110	40.067	2.004	1.616	2.484
**Work Balance**						
STRESS	−0.308^***^	0.086	12.808	0.735	0.621	0.870
**Self-Efficacy and Work-Related Behavior**						
PERSISTENCE	0.112^*^	0.061	3.295	1.118	0.991	1.261
OVERCAME	0.338^***^	0.092	13.419	1.403	1.170	1.681
CALM	−0.211^**^	0.072	8.500	0.810	0.703	0.933
**Hardy Personality and Work-Related Behavior**						
CONSCIENTIOUSNESS	0.447^***^	0.146	9.371	1.564	1.175	2.083
NEXPERIENCE	0.415^**^	0.155	7.163	1.514	1.117	2.052
INNOVATIVENESS	0.284^*^	0.139	4.144	1.328	1.011	1.745
COMMITMENT	0.622^***^	0.136	21.062	1.862	1.428	2.429
**EMPLOYEES IN PAID ONLINE TRAINING (*****N*** = **461)**						
Constant	−18.647^***^	1.930	91.513	–	–	–
**Job Ambition**						
TRAINING	0.239^N.S.^	0.163	2.149	1.270	0.923	1.749
CORRECTING	0.574^***^	0.178	10.420	1.776	1.253	2.518
NTRENDING	0.307^*^	0.159	3.718	1.359	0.995	1.856
PERSEMPOWR	0.908^***^	0.181	25.118	2.480	1.739	3.538
**Organizational Identification**						
PARTICIPATION	0.697^***^	0.153	20.877	2.008	1.489	2.708
**Work Balance**						
STRESS	−0.254^*^	0.123	4.246	0.776	0.609	0.988
**Self-Efficacy and Worked-Related Behavior**						
PERSISTENCE	0.137^N.S^	0.090	2.317	1.146	0.961	1.367
OVERCAME	0.313^**^	0.128	5.939	1.367	1.063	1.758
CALM	−0.100^N.S^	0.100	0.997	0.905	0.744	1.101
**Hardy Personality and Work-Related Behavior**						
CONSCIENTIOUSNESS	0.561^**^	0.219	6.572	1.753	1.141	2.693
NEXPERIENCE	0.630^**^	0.225	7.834	1.878	1.208	2.919
INNOVATIVENESS	0.250^N.S^	0.193	1.673	1.284	0.879	1.875
COMMITMENT	0.620^***^	0.198	9.809	1.858	1.261	2.738

*** p < 0.01;

** p < 0.05;

* *p < 0.1; N.S., Not significant*.

The dataset allows our sample to be segmented by employee online training purchase. In this regard, we have segmented our microdata into two samples: employees involved in paid online training activities (*N* = 461) and otherwise (*N* = 422). With the first sample of employees, we replicated the explanatory model of greater predisposition toward employability. The results are also shown in Table 5.

Regarding employees in paid online training, the probabilistic model correctly classified 82.4% of employees (79.3% of those that do not have a greater disposition toward employability and 85.2% of those that have a greater disposition toward employability). The Cox-Snell and Nagelkerke *R*^2^-values were 0.44 and 0.59, respectively. The improvement (from 637.5 to 869.4) in the likelihood function is significant. It confirms the goodness-of-fit of the predictive capacity, and the variables as a whole have an outstanding explanatory power (Chi-square Hosmer-Lemeshow test = 11.8; *p* = 0.049).

Comparing standardized coefficients and odds ratios [Exp (β)] between all employees and employees in online training, the second ones attribute greater explanatory power of dispositional employability to career-related personal empowerment, new experiences related to the job and conscientiousness in the preparation for positive job outcomes. In this regard, the effect of online training on predisposition toward employability would be indirect. Being involved in paid online training activities is not a direct explanatory factor of dispositional employability. However, certain types of behavior of employees participating in paid online training would determine a positive effect on greater dispositional employability. This is especially the case for those most sensitive to career-related personal empowerment who are more predisposed to developing new experiences at work and more aware of the fact that positive job outcomes are related to preparation conscientiousness.

### Online training purchase explanatory factors

The probabilistic model correctly classified 74.2% of employees (69.7% of those that do not purchase online training and 78.5% of those that purchase online training). The Cox-Snell and Nagelkerke *R*^2^-values were 0.33 and 0.37, respectively. The improvement (from 400.5 to 1105.3) in the likelihood function is significant. It confirms the goodness-of-fit of the predictive capacity, and the variables as a whole have an outstanding explanatory power (Chi-square Hosmer-Lemeshow test = 16.2; *p* = 0.037).

From the estimation of the model (Table 6), it was found that all the variables included have significant explanatory power for Spanish employees' online training purchase (*p* < 0.1 in the worst case). Regarding standardized coefficients and odds ratios [Exp (β)], it is possible to assess the considerations referred to below.

**Table 6:** **Determinants of the online training purchase**.

	**Standardized coefficient**	**Standard error**	**Wald**	**Exp (β)**	**95% CI**	
					**Lower**	**Higher**
Constant	−0.230^***^	0.627	4.135	–	–	–
**SOCIO-DEMOGRAPHIC CONDITIONS**						
AGE	−0.350^***^	0.084	17.583	0.704	0.598	0.830
GENDER	−0.208^*^	0.145	2.061	0.812	0.611	0.979
**DISPOSITIONAL EMPLOYABILITY FACTORS**						
MOTIVATION	0.270^*^	0.156	2.984	1.310	1.004	1.779
IDENTITY	0.341^**^	0.149	5.208	1.406	1.049	1.884
**JOB AMBITION**						
TRAINING	0.321^***^	0.083	14.793	1.379	1.117	1.624
NTRENDING	−0.302^***^	0.084	12.854	0.739	0.626	0.872
**SELF-EFFICACY AND WORK-RELATED BEHAVIOR**						
CALM	−0.175^***^	0.047	13.789	0.839	0.765	0.921
EFFORT	0.283^***^	0.095	8.871	1.327	1.102	1.598
CHALLENGE	0.349^***^	0.126	7.711	1.418	1.108	1.814
VARIETY	0.392^***^	0.119	10.923	1.480	1.173	1.867
NOVELTY	0.377^***^	0.117	10.330	1.350	1.192	1.672

*** p < 0.01;

** p < 0.05;

* *p < 0.1; N = 883*.

First, it was confirmed that those most likely to purchase online training are young employees followed by female employees. Second, the relationship between dispositional employability and online training purchase is not direct. The dichotomous composite indicator of dispositional employability is not significant in the model. However, employees who have certain dispositional employability factors tend to do more online training. These particular predispositions are related to motivation, i.e., optimism and control over career opportunities, and assigning and planning career goals; and to work identity, i.e., the importance of success and external acknowledgment of work. And third, it was also found that the employees' job-related behavior also determines online training purchase. This behavior is associated with employees' job ambition, self-efficacy, and hardy personality. Specifically, it was found that employees who spend more time on training and have less contact with new trends in their jobs, find it hard to keep calm in difficult situations, and have a greater predisposition toward effort, and preference for novelty, variety, and challenges at work are more likely to purchase online training.

## Discussion

Employability, understood as an individual's ability to get and keep a job, is a synergistic collection of characteristics driven by employees' motivation and behavior. Although prior education and training play a decisive role as predictors of employability, the findings in the literature on career success have tempered this relationship. In this sense, new research highlights the importance of personality characteristics and individuals' behavior as determinants of not only academic performance but also job outcomes. As a result of this evidence, the literature shows that a dispositional employability approach has been developed, which focuses on the perception of individuals' motivational attitudes and behavior related to careers and work in general. Also, the flexibilization, segmentation, and individualization of labor markets, the advent of new work organization and human resources practices, and the global knowledge-based economy have fostered new approaches to employability research. Again, the literature highlights the relevance of motivational attitudes toward new job-related trends.

Online training acquires special relevance in the sense that continued learning is placed at the heart of career development and employees' improved employability. Indeed, and despite its significant potential, empirical evidence has now tempered the online training effects on employability. Little attention to students' specific characteristics and behavior, such as attitude toward careers, seems to explain the weak results of online training for employability. Finally, and beyond their technological and pedagogical dimensions, employees' personal, and motivational determinants of online training enrolment and satisfaction have also been addressed in the literature.

Through a sample of 833 employees working for enterprises in Spain, our research proposed three main objectives: to construct a dispositional employability indicator, to analyze the relationship between online training and dispositional employability, and to study the effect of dispositional employability on online training purchase.

### Dispositional employability evidence and implications

Using principal component exploratory analysis, dispositional employability was found to be characterized by five factors comprising 23 items: “openness to changes at work,” “career motivation and work resilience,” “work and career proactivity,” “optimism and engagement at work,” and “work identity.” The results obtained for our sample of Spanish employees are consistent with previous research (Fugate and Kinicki, 2008). The indicator presented here has practical value both for managers (especially in human resources practices) and for employees (especially for improving their career development).

Using logit binomial regression analysis, the research obtained the motivational factors explaining dispositional employability. Specifically, it is concluded that employees who are more motivated to anticipate and optimize job changes, who identify with and are involved in corporate sense, who have less stress, who have self-efficacy or the ability to manage obstacles, overcome difficulties and achieve their goals, and who have hardy personality traits to commit, challenge, and control job situations are more likely to have greater dispositional employability. This approach seems to be appropriate for detecting individual differences and to tailor interventions. An assessment based on dispositions can be used to identify personal strengths and weaknesses that should be accounted for in future careers, which may help to develop an individualized coaching trajectory (Vanhercke et al., 2014). Indeed, our evidence suggests that employability motivations could be linked with employability outcomes, but this relationship must be tested in the future.

Furthermore, it seems reasonable to suggest that employees with higher dispositional employability are well suited to empowered, supportive, and developmental management and work practices, due to their proactive and active motivations (Fugate and Kinicki, 2008). In knowledge-based work environments, the importance of employees' future job expectations has been highlighted (Gallie et al., 2012; Graso and Probst, 2012) in terms of explaining employability improvements. But, the link between employee perceptions of control and coping with labor and organizational change must be analyzed in the future.

### Online training and dispositional employability evidence and implications

Using logit binomial regression analysis, the research also obtained the relationship between dispositional employability and online training. Consistent with suggestions in the literature (Inayat et al., 2013; Kyndt and Baert, 2013; Silva et al., 2013), for the whole sample of employees we did not obtain any direct relationship. In other words, employees' involvement in online training does not explain *per se* motivational predisposition toward employability. To observe the effect of online training on dispositional employability, we had to analyze a specific sample of employees in online training, and address their motivations and behaviors. Our results suggest that employees participating in online training who are more sensitive to career-related personal empowerment, more predisposed to developing new experiences at work, and more aware of the fact that positive job outcomes are related to preparation conscientiousness are more likely to have greater dispositional employability.

These results are consistent with the latest evidence on workplace learning outcomes (Virtanen et al., 2014). “Active membership” (i.e., influencing the way things were done at the workplace), “invention” (students' willingness to invent new solutions at work), and “learning orientations” (students' willingness or motivation to learn new things at work) are also determinants in non-online workplace learning environments. Despite this evidence, further investigations need to be conducted in the future. Particular attention should be paid to the relationship between employee-related motivational factors (both in online and non-online training environments), learning design and job-related structural features.

Finally, and using binomial logit analysis, we obtained the socio-demographic, dispositional employability, and motivational factors explaining online training purchase. Employees are more likely to purchase online training if they are younger, women, more motivated, and better identify with their jobs, have more time to spend on training, do not have recent contact with new trends in their jobs, find it hard to keep calm in difficult situations, have a greater predisposition toward effort, and preference for novelty, variety, and challenges at work.

One of the relevant implications of this is that overall dispositional employability does not explain *per se* online training purchase. We only found significant effects for two specific motivational factors: the first factor relates to optimism and control over career opportunities, and assigning and planning career goals; and the second factor refers to work identity. On the other hand, job ambition (Van der Heijde and Van der Heijden, 2006), self-efficacy (Baessler and Schwarcer, 1996), and hardy personality (Moreno-Jiménez et al., 2001) employee-related motivational effects are also obtained. Self-initiating proactive job ambition entails employees' preparing for future work changes in order to strive for the best possible job and career outcomes. Self-efficacy refers to employees' ability to appropriately manage a wide range of obstacles, overcome difficulties and achieve their goals. Employees with hardy personalities are characterized by a high degree of commitment, experiencing situations as challenges rather than as threats, and tend to perceive certain internal control in various situations. Therefore, we have linked a set of employees' motivational and behavioral factors explaining online training purchase. But, as pointed out in the literature (Brown, 2005; Rovai et al., 2007; Chiu and Wang, 2008; Paechter et al., 2010), motivational factors interrelate with other personal, structural, learning, and social determinants in explaining online training purchase. In this regard, future research on interaction effects should be conducted.

Nevertheless, our contribution is relevant for online training organizations. Despite the rise in online training enrolment, competition in the supply of e-learning programs (formal and informal) is growing exponentially. E-learning organizations increasingly need to take new approaches toward their consumers, and affective loyalty plays a greater role in explaining the rise in market shares. Knowing about the motivations and behavior of employees (students) that buy online products is a strategic option that should be considered in markets with ever-increasing competition. In the future, extension in terms of time and sample size will allow us to further our knowledge of how the motivational and behavioral effects explain online training purchase.

## Author contributions

All the authors made substantial contributions to the design, data analysis, and interpretation of the results. JT contributed to the formulation of the research questions, study design, literature review, interpretation of the results, and article drafting. He is the corresponding author and the guarantor of the article. PF participated in the formulation of the research questions, study design, literature review, data analysis, statistical modeling, interpretation of the results, and article drafting. JB participated in the data analysis and statistical modeling. All the authors have read, revised, and approved the final manuscript.

### Conflict of interest statement

The authors declare that the research was conducted in the absence of any commercial or financial relationships that could be construed as a potential conflict of interest.
